# Insecticide tolerance of the malaria vector *Anopheles gambiae* following larval exposure to microplastics and insecticide

**DOI:** 10.1371/journal.pone.0315042

**Published:** 2024-12-12

**Authors:** Dativa J. Shilla, Deokary Joseph Matiya, Nyanda Laini Nyamandito, Mgeni Mohamed Tambwe, Richard S. Quilliam

**Affiliations:** 1 Dar es Salaam University College of Education, University of Dar es Salaam, Dar es Salaam, Tanzania; 2 Dar es Salaam Institute of Technology, Dar es Salaam, Tanzania; 3 Ifakara Health Institute, Bagamoyo, Tanzania; 4 Biological and Environmental Sciences, Faculty of Natural Sciences, University of Stirling, Stirling, United Kingdom; Universidade Federal do Rio de Janeiro, BRAZIL

## Abstract

Microplastic (MP) pollution poses a global threat to urban and rural environments and can have negative effects on a range of organisms. Mosquito larvae often breed in water contaminated with MPs, and given their important role as disease vectors, understanding the effects of larval exposure to MPs is critical for understanding the potential impact on their life history traits and subsequent methods for their control. Here, we have exposed first instar larvae of *Anopheles gambiae* s.s. to environmentally realistic concentrations of PET microplastics (1.0–7.5 μm) and a sub-lethal dose of insecticide mixed with microplastics, and quantified survival, development, and susceptibility of larvae over six generations. Adult mosquitoes from larvae exposed to these treatments were subsequently tested for insecticide resistance. Exposure to MPs decreased larval survival rates compared to the control; however, over six generations of exposure, survival rates significantly increased. Similarly, there was a higher survival rate of those larvae exposed to MPs mixed with insecticide compared to those exposed to just the insecticide, and survival increased further over the six generations. For the adult mosquito susceptibility tests, knockdown times (KDTs) indicated some level of insecticide tolerance when larvae had been previously exposed to MPs and insecticides. This is the first study demonstrating the selection of insecticide tolerance in adult mosquitoes after consecutive generations of larval exposures to varying concentrations of MPs. Therefore, field-scale studies are now urgently required to quantify whether larval insecticides are less effective at controlling mosquitoes in breeding sites commonly polluted with MPs.

## Introduction

Plastic pollution in the environment is increasing due to the continuous production, consumption, and inadequate or inappropriate disposal of plastic residues [1, 2]. Discarded plastic wastes can have indirect effects, e.g., by acting as containers for rainwater accumulation and thus, providing breeding habitat for [3–5]; with the potential for this increased habitat to facilitate the emergence of mosquito-borne diseases such as malaria, dengue and chikungunya [6]. Plastic pollutants are categorised according to their size, shape, and composition, but once in the environment, plastic particles of all sizes and polymers can become fragmented into smaller pieces [7, 8].

Mosquitoes undergo complete metamorphosis, transitioning through various aquatic and terrestrial stages in their life cycle. Their larvae typically thrive in permanent and temporary freshwater habitats, including puddles, rice paddies, and road ruts [9]. Such breeding sites, are often contaminated with toxic organic and inorganic pollutants, and microplastics (< 5mm) originating from wastewater, agricultural activities, sewage sludge disposal, landfill, and industrial effluents [10]. As non-selective filter feeders, mosquito larvae primarily consume microorganisms, algae, and organic debris, but can also unintentionally ingest microplastics (MPs) [11]. Exposure to polyethylene microplastic particles can affect survival of larvae of *Aedes albopictus* [12], whilst exposure to polyethylene or polystyrene microplastics can alter the developmental rate of *Culex quinquefasciatus* [13]. MPs can also act as carriers for other contaminants, including persistent organic pollutants (POPs) and heavy metals [14, 15], and the interactive effects of MPs on mosquito larvae could impact their ability to transmit pathogens [16], and potentially alter their adaptability and density in areas susceptible to mosquito-borne diseases.

Prolonged exposure to MPs may lead to biological adaptation, and favour individuals better equipped to thrive in such environments (e.g., Khosrovyan et al. [17]. This adaptation to MPs could also impact responses to other chemicals, including larvicides used for controlling mosquitoes, for example, *Aedes aegypti* mosquitoes exposed to a range of environmental pollutants over six generations showed accelerated development and increased tolerance to the bioinsecticide *Bacillus thuringiensis israelensis* (Bti) [18]. Importantly, MPs have the potential to sorb insecticides and other organic pollutants in aquatic environments due to their intrinsic properties and large surface area [19, 20]. This retention of insecticides by MPs in water containing mosquito larvae may reduce the effectiveness of larvicidal insecticides in mosquito breeding sites by strongly binding the insecticide and making it unavailable; however, depending on the specific interaction and dissociation potential of larvicides with different MP polymers, some MPs may be capable of delivering insecticides directly to mosquito larvae.

In this study, polyethylene terephthalate (PET) MPs were selected for experimentation due to their pervasiveness in the environment and widespread use in textile fibers, consumer goods packaging, and bottles [21, 22]. PET-MPs are known to exert harmful effects on certain insect species [22–24]. However, to date, no research has investigated the potential effects of PET-MPs on mosquitoes, or the cumulative effect of PET-MPs and insecticides; therefore, we quantify the effect of exposing larvae to a mixture of MPs and lambda-cyhalothrin on subsequent insecticide tolerance in adult mosquitoes. Lambda cyhalothrin is a cost-effective pyrethroid insecticide widely used in vector control strategies, including indoor residual spraying (IRS), insecticide-treated bed nets (ITNs), and space spraying [25]. Moreover, although lambda-cyhalothrin is primarily used as an adulticide, it also exhibits larvicidal activity and has the ability to adsorb onto microplastics [26], a property similarly observed in larvicides like pyriproxyfen [27].

Evolving insecticide resistance in malaria vectors is a significant global challenge [28], and given the increasing prevalence of MPs in the environment, this study aimed to test the hypothesis that exposure to MPs could reduce the efficacy of insecticide to *An*. *gambiae* s.s. Specifically, our objectives were to expose the first instar larvae of *An*. *gambiae* s.s. to PET-MPs both independently and in a mixture with a sub-leathal dose of lambda-cyhalothrin over six generations, and (1) quantify the impact on larval survival rate and development time; and (2) quantify subsequent effects on adult *An*. *gambiae* s.s. grown from larvae exposed to PET-MPs and mixtures of PET-MPs and insecticide.

## Methods and materials

### Mosquito rearing

The study used a laboratory colony of *An*. *gambiae* s.s. (Ifakara strain) from the Ifakara Health Institute (IHI) in Bagamoyo, Tanzania, which is fully susceptible to all commonly used insecticide classes. This choice of *Anopheles* species is based on their wide-ranging distribution and their importance to public health in sub-Saharan Africa. Aquatic stages (larvae and pupae) of stock colonies were reared in controlled environment conditions at a temperature of 30 ± 2℃, and 80 ± 10% relative humidity, under a 12h photoperiod. Once eggs were hatched, the larvae were transferred to deionised water in 2L ceramic bowls (Shenzhen Tao Hui Industrial Co., Ltd) and fed daily with 0.18 g/L of dried fish feed (Tetra, UK). The emerged adults were reared in net cages in standard insectary conditions (as above). The adult mosquitoes were fed with a 10% sucrose solution through filter paper. In preparation for egg development, female mosquitoes were fed with cow blood using a membrane-feeding system [29].

### Polyethylene terephthalate (PET) MPs

PET-MP fragments with a width range of 1.0–7.5 μm and density of 1.38g/cc were purchased from Cospheric LLC (Santa Barbara, USA). A stock solution (100 mg/20 mL) was stored at 4°C in the dark. To minimize aggregation, the solution was shaken (150 rpm) for an hour before subsequent dilution into three different concentrations (0.1 g/L, 1 g/L and 10 g/L).

### Assessment of larval survival and development time

Experimental exposure was conducted in an environmentally-controlled insectary room at Ifakara Health Institute. Eight treatments were used (Table 1) with each treatment having six replicates, and each replicate consisting of 60 larvae. Pre-selection of a lethal concentration (0.140 x 10^-10^g/l) of the insecticide lambda-cyhalothrin (Karate) (AGRO SHOPY-Syngenta India LTD) was conducted to establish a sub-lethal dose (LD_15_) that would kill at least 15% of the tested larvae. We avoided higher doses of the insecticide, such as LD_50_, because prolonged exposure to these doses throughout larval development leads to significant larval mortality. The control group was prepared using deionised water alone while treatments with MPs alone were prepared in deionized water at three different concentrations (0.1, 1.0, and 10 g/L). Mixture treatments, were prepared in deionized water by mixing MPs (0.1, 1.0 and 10 g/L) and lambda-cyhalothrin (0.140 x 10^-10^g/l). Importantly, we have used a conservative dose of microplastics, i.e., lower than environmental concentrations recorded from replicate water samples collected from the Msimbazi River (a malaria vector larval habitat) in Dar es Salaam, which ranged from 240–2300 particles/L (unpublished data).

**Table 1:** Each treatment comprised six replicates, with each replicate containing sixty larvae. Larvae from each generation (G1-G6) were exposed to the same treatment.

Treatment	PET-MP	Insecticide^*^
1	-	-
2	0.1 g/L	-
3	1.0 g/L	-
4	10 g/L	-
5	-	+
6	0.1 g/L	+
7	1.0 g/L	+
8	10 g/L	+

*0.140 x 10^−10^ g/L

This experiment followed WHO guidelines, requiring four or more replicates with approximately 25 larvae per replicate [30]. We used a total of 14,000 larvae across eight treatments: 3 MP concentrations, 3 mixtures, 1 control, and 1 insecticide solution, spanning six generations (Table 1). Each generation included six replicates per treatment with 60 larvae each, resulting in 300 larvae per treatment and 2,400 larvae per generation. Each replicate was treated in a separte 500 ml ceramic mesocosm (Shenzhen Tao Hui Industrial Co., Ltd). MP suspensions were added to each experimental mesocosm and measurements recorded daily. Developed pupae were collected using a dropper and put into labelled net cages for the emergence of adult mosquitoes. The emerged adults were fed through filter papers soaked in 10% sucrose solution, and for egg development, female mosquitoes were fed with cow blood using a membrane-feeding system. Larvae were taken through six larvae-adult generations (G1 –G6), with the larvae exposed to the same treatment at each generation. G0 (baseline generation) were not exposed to any of the treatments and provide an unexposed control.

### Insecticide susceptibility of adult female mosquitoes

This study assessed susceptibility across three generations (G0 [non-exposed mosquitoes], G3, and G6) following WHO guidelines [31]. These generations were selected to monitor the progression of tolerance over the exposure period. For the G0 generation, 100 adult female mosquitoes were divided into four replicates of 25 each, along with a control group of 25. This experiment was conducted twice, resulting in a total of 200 test mosquitoes and 50 controls. In the G3 generation, 100 adult female mosquitoes were divided into four replicates and tested across eight treatment conditions: control, insecticide, 0.1 g/L microplastics (MPs), 1 g/L MPs, 10 g/L MPs, 0.1 g/L mixture, 1 g/L mixture, and 10 g/L mixture, each with a corresponding control group. This setup was repeated twice, yielding a total of 1,600 test mosquitoes and 400 controls for the G3 generation. The same experimental design was repeated during the G6 generation, resulting in 1,600 test mosquitoes and 400 controls

Emerged adult female mosquitoes were aspirated from respective cages, and placed into resting tubes for one hour of acclimatization. They were subsequently transferred into WHO testing tubes lined with 0.05% lambda-cyhalothrin-impregnated paper (Manufactured by University Sains, Penang, and Malaysia, on behalf of the WHO). Two testing groups were transferred to insecticide-free testing tubes as control groups. The knockdown time (paralysis within 60 minutes of exposure) and mortality rate (death after 24 hours of exposure) are considered essential parameters for testing the resistance of mosquitoes to insecticides. The knockdown time (KDT) was recorded at the following time intervals: 5, 10, 15, 20, 30, 40, 50, and 60 minutes. After insecticide exposure, mosquitoes were transferred back into resting tubes and fed on a 10% sucrose solution. The mortality rate was recorded after 24 hours of resting time. The testing environment had temperature and humidity between 24 to 28^˚^C and 68 to 74%, respectively. Based on the WHO resistance report criteria [31], after 24 h post-exposure to insecticides mosquitoes are considered to be ‘susceptible’ when mortality is between 98% up to 100%, ‘possibly resistance’ when mortality is 90% up to 97%, and ‘confirmed resistance’ when mortality is below 90%.

### Statistical analysis

Prior to statistical analysis, data were assessed for normality using a Q-Q plot of residuals, which is an appropriate method for large sample sizes [32, 33]. Once the data were confirmed to follow a normal distribution, variations in mean survival rates and development times across different treatments and generations of mosquito larvae were analyzed using a univariate general linear model (GLM). Pairwise multiple comparisons were Bonferroni-adjusted, with analyses conducted using SPSS software (IBM SPSS Statistics v. 22). Additionally, Kaplan-Meier survival curves were generated to illustrate the trend of larval survival across generations.

For the susceptibility tests, the percentage of female adult mosquitoes knocked down over time was analyzed using regression via probit analysis (IBM SPSS Statistics v. 22). This method was used to determine the time in minutes required for 50% (KDT_50_) and 95% (KDT_95_) of the adult mosquitoes to be knocked down in the G0, control, G3, and G6 groups. To quantify resistance, the resistance ratio was calculated by dividing the KDT50 of each treated group (G3 or G6) by the KDT50 of the untreated control group (G0), i.e., the Resistance Ratio = KDT_50_ in each treatment for G3 or G6) / (KDT_50_ for G0). This ratio offers a comparative measure of knockdown efficacy between treated and untreated populations, indicating the level of resistance. The mean mortality rates of mosquitoes, recorded after 24 hours of holding time, were also analyzed using a GLM, with Bonferroni-adjusted pairwise multiple comparisons.

## Results

### Impact of MPs and mixture treatments on larvae survival rate

**Fig 1:**
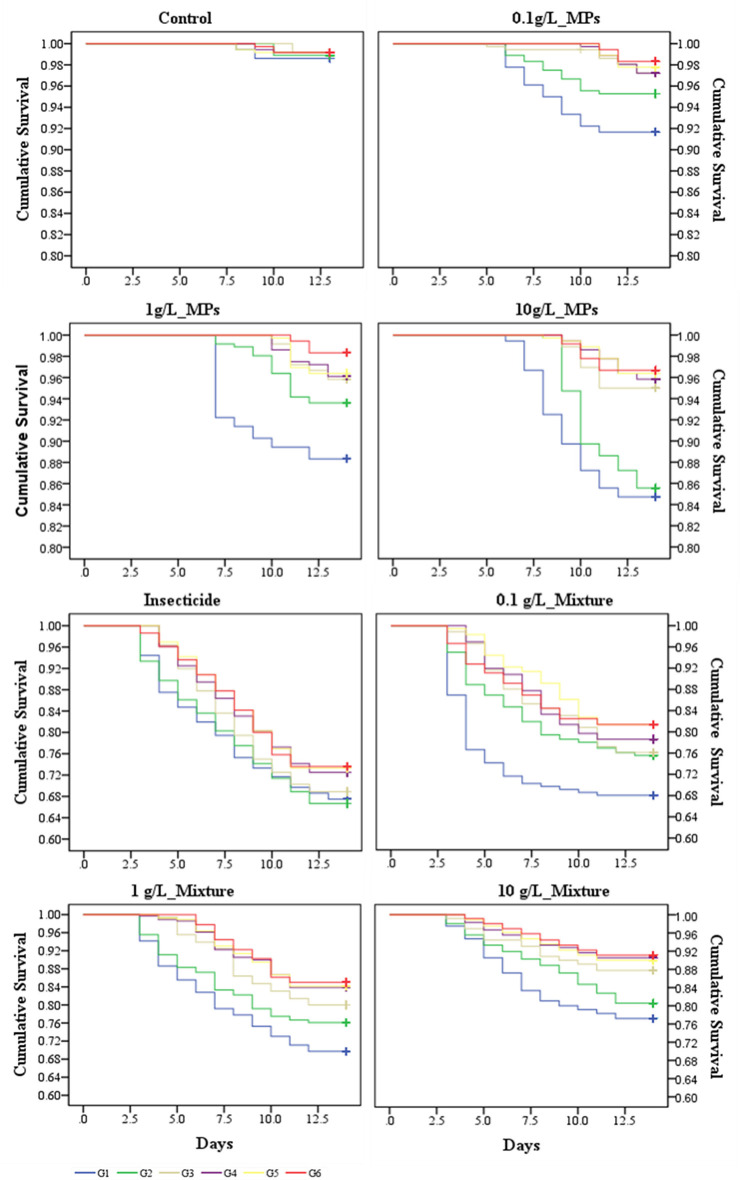
Survival curves of larvae exposed to MPs or a mixture of MPs with insecticide. The curves show the cumulative survival of six successive generations of larvae following exposure to one of the eight treatments (note different y axis scales).

Survival rates for each generation significantly decreased across treatments containing only MPs (F₍₃,₁₄₄₎ = 81.6, p < 0.0001) (Table 2), with mean survival rates decreasing from 91.7–98.4% at 0.1 g/L to 84.6–96.8% at 10 g/L. These survival rates were significantly lower than those of the control group (98–99.3%) (p < 0.0001) (Table 3). However, across generations, the average survival rate significantly increased from generation 1 (G1) (84.6–91.7%) to generation 6 (G6) (96.8–98.4%) (F₍₅,₁₄₄₎ = 55.3, p < 0.0001), as shown by the survival curves (Fig 1). Survival rates were significantly higher from generation 2 (G2) to generation 6 (G6) compared to G1 (p < 0.0001), with no significant differences observed between generations G3, G4, G5, and G6 (p > 0.05) (Table 3).

**Table 2:** Mean survival rate of larvae exposed to MPs and MPs mixed with insecticide, over six generations. Values are the mean of 6 replicates ± standard deviation.

Treatment	Mean survival rate (%)					
	G1	G2	G3	G4	G5	G6
Control	98.0 ± 2.2	99.0 ± 2.0	99.3 ± 1.6	99.3 ± 1.4	99.3 ± 1.7	99.3 ± 1.4
0.1 g/l MPs	91.7 ± 8.6	95.3 ± 6.2	97.6 ± 4.6	97.6 ± 4.2	97.9 ± 2.3	98.4 ± 6.7
1.0 g/l MPs	88.2 ± 11.5	93.5 ± 8.5	95.7 ± 5.4	96.0 ± 6.3	96.5 ± 3.4	98.4 ± 4.3
10 g/l MPs	84.6 ± 13.5	85.6 ± 12.8	95.1 ± 6.4	95.7 ± 7.3	96.3 ± 6.1	96.8 ± 7.1
Insecticide	67.5 ± 16.8	66.8 ± 17.9	68.9 ± 18.0	72.5 ± 17.9	73.4 ± 15.8	73.5 ± 11.7
0.1 g/l Mixture	68.0 ± 15.8	75.6 ± 12.6	76.0 ± 14.9	78.7 ± 16.1	81.3 ± 13.4	81.5 ± 11.0
1.0 g/l Mixture	69.8 ± 14.0	76.1 ± 12.4	80.0 ± 15.5	83.8 ± 13.3	84.3 ± 12.6	85.0 ± 8.7
10 g/l Mixture	77.3 ± 13.6	80.6 ± 12.1	87.9 ± 6.1	90.5 ± 6.2	90 ± 11.5	91.1 ± 6.9

**Table 3:** Bonferroni-adjusted multiple comparisons of the mean survival rate of *An*. *gambiae* s.s. across generations and treatments.

Posthoc Tests Across Generations				Posthoc Tests Across Treatments		
		P value				
Generation vs Generation		MPs	Mixture	Treatment vs Treatment		P- value
G1	G2	< 0.0001	< 0.0001	***MPs***		
	G3	< 0.0001	< 0.0001	Control	0.1g/L MPs	< 0.0001
	G4	< 0.0001	< 0.0001		1g/L MPs	< 0.0001
	G5	< 0.0001	< 0.0001		10g/L MPs	< 0.0001
	G6	< 0.0001	< 0.0001	0.1g/L MPs	1g/L MPs	< 0.0001
G2	G3	< 0.0001	< 0.0001		10g/L MPs	< 0.0001
	G4	< 0.0001	< 0.0001	1g/L MPs	10g/L MPs	0.001
	G5	< 0.0001	< 0.0001	***Mixture***		
	G6	< 0.0001	< 0.0001	Insecticide	0.1 g/L_Mixture	< 0.0001
G3	G4	1	1		1g/L_Mixture	< 0.0001
	G5	1	0.508		10g/L_Mixture	< 0.0001
	G6	1	0.152	0.1 g/L Mixture	1g/L_Mixture	< 0.0001
G4	G5	0.311	1		10g/L_Mixture	< 0.0001
	G6	1	1	1 g/L Mixture	10g/L_Mixture	< 0.0001
G5	G6	0.508	1			

The average survival rate of larvae exposed to the mixture of MPs and insecticide (referred to as the "mixture") significantly increased from 68–81.5% at 0.1 g/L to 77.3–91.1% at 10 g/L (F₍₃,₁₄₄₎ = 115.8, p < 0.0001) (Table 2). Survival rates were significantly higher in all mixture treatments compared to the insecticide-only treatment (67.5–73.5%) (p < 0.0001) (Table 3). Across generations, survival rates in each mixture treatment significantly increased from G1 (68–77.3%) to G6 (81.5–91.1%) (F₍₅,₁₄₄₎ = 44.3, p < 0.0001) (Fig 1). Survival rates were significantly higher from G2 to G6 compared to G1 (p < 0.0001), and from G3 to G6 compared to G2, with no significant differences between G3 and subsequent generations (p > 0.05). However, larvae exposed to MPs alone consistently exhibited higher survival rates compared to those exposed to the mixture of MPs and insecticide (p < 0.0001).

### Impact of MPs and MPs mixed with insecticide on larval development

The overall mean development time of larvae (defined as the duration from larval stage to pupation), was significantly prolonged across varying concentrations of MPs treatments (F₍₃,₇₇₄₎ = 31.2, p < 0.0001). Specifically, the development time increased from 9.8–11.6 days for larvae exposed to a concentration of 0.1 g/L MPs to 11.2–12.6 days for larvae exposed to a concentration of 10 g/L (Table 4). The mean development time in the MP treatments was significantly greater than that observed in the control group, which ranged from 9.8 to 10 days (p < 0.0001). Furthermore, higher concentrations of MPs resulted in longer development times compared to lower concentrations (p < 0.0001) (Table 5). Conversely, the mean development time exhibited a distinct trend with significant variation across generations (F₍₅,₈₇₂₎ = 7.97, p < 0.0001). Specifically, the mean development time increased from G1 to G4 before decreasing in G5 and G6 for all MPs treatments, with significantly higher development time observed in G4 than in G1 and G6 (p < 0.0001).

**Table 4:** Mean development time of larvae exposed to MPs and mixtures of MPs with insecticide, over six generations.

	Development time (days)					
	G1	G2	G3	G4	G5	G6
Control	9.8 ± 1.7	10.0 ± 1.9	10.0 ± 2.0	10.0± 1.8	10.0 ± 1.6	9.8 ± 1.7
0.1 g/l MPs	9.8 ± 1.5	10.7 ± 1.6	11.1 ± 1.9	11.6 ± 2.1	11.2 ± 1.0	11.1 ± 1.6
1.0 g/l MPs	10.2 ± 1.3	10.7 ± 1.7	11.1 ± 1.9	11.7 ± 2.0	11.5 ± 0.8	11.3 ± 1.6
10 g/l MPs	11.4 ± 1.5	11.2 ± 1.4	11.3 ± 1.4	12.6 ± 1.7	11.6 ± 1.0	11.4 ± 1.8
Insecticide only	11.3 ± 1.9	11.4 ± 2.0	13.7 ± 1.9	12.9 ± 2.4	12.7 ± 1.9	12.3 ± 2.2
0.1 g/l Mixture	11.2 ± 1.3	11.2 ± 1.8	12.6 ± 2.2	12.6± 1.8	12.2 ± 1.5	12.0 ± 2.4
1.0 g/l Mixture	11.1 ± 2.2	11.1 ± 2.1	11.6 ± 2.3	12.5 ± 1.9	11.9 ± 1.4	11.7 ± 1.9
10 g/l Mixture	10.5 ± 2.1	10.8 ± 2.0	11.5 ± 2.3	11.7 ± 1.6	11.4 ± 1.8	11.2 ± 1.6

Values are the mean of 6 replicates ± standard deviation

**Table 5:** Bonferroni-adjusted multiple comparisons of the development time of larvae exposed to MPs and mixture treatment.

Posthoc Tests Across Generations				Posthoc Tests Across Treatments		
*Generation vs Generation*		*P value*		*Treatment vs Treatment*		*P value*
		*MPs*	*Mixture*			
G1	G2	1	0.006	***MPs***		
	G3	0.063	< 0.0001	Control	0.1g/L MPs	<0.0001
	G4	<0.0001	< 0.0001		1g/L MPs	<0.0001
	G5	0.005	< 0.0001		10g/L MPs	<0.0001
	G6	0.983	1	0.1g/L MPs	1g/L MPs	1
G2	G3	1	0.019		10g/L MPs	0.001
	G4	0.001	< 0.0001	1g/L MPs	10g/L MPs	0.001
	G5	0.772	< 0.0001	***Mixture***		
	G6	1	< 0.0001	Insecticide	0.1 g/LMixture	0.115
G3	G4	0.044	1		1 g/L_Mixture	< 0.0001
	G5	1	1		10 g/L_Mixture	< 0.0001
	G6	1	0.105	0.1 g/L Mixture	1 g/L_Mixture	0.461
G4	G5	1	1		10 g/L_Mixture	< 0.0001
	G6	<0.0001	0.04	1 g/ Mixture	10 g/L_Mixture	0.047
G5	G6	0.597	1			

In larvae exposed to the mixture treatments the development time consistently decreased from 11.2–12.6 days at the lowest concentration (0.1 g/L,) to 10.5–11.7 days at the highest concentration (10 g/L,) (F₍₃,₇₇₄₎ = 16.98, p < 0.0001). However, larvae exposed to both the 1 g/L and 10 g/L mixture treatments showed significantly shorter development times compared to larve exposed to just the insecticide-only treatment (11.5–12.9 days) (p < 0.0001). (Table 4). The mean development time of larvae increased from G1 to G4, before decreasing in G5 and G6, with a significantly higher development time in G4 than in G1, G2, and G6 (p < 0.0001) (Table 5).

### Insecticide susceptibility of adult female *An*. *gambiae* s.s

#### knockdown time (KDT_50_ and KDT_95_)

At G0 (baseline), the KDT_50_ and KDT_95_ were 17.4 and 32 minutes, respectively (Table 6). By G3 in treatments containing just MPs, the KDT_50_ ranged from 19.7 to 31.7 minutes, and the KDT_95_ from 32 to 62 minutes. Similar trends were observed in G6, with KDT_50_ ranging from 21 to 35 minutes and KDT_95_ from 28 to 70 minutes. Resistance ratios, obtained by dividing the KDT_50_ of either G3 or G6 by that of G0, ranged from 1 to 1.8 in G3 and from 1 to 2 in G6 across various treatments.

**Table 6:** Knockdown time (KDT_50_ and KDT_95_) in minutes of adult female *An*. *gambiea s*.*s* after 60 minutes insecticide exposure.

Generation	Treatment	KDT_50_ (95%CI)	KDT_95_ (95%CI)	Resistance ratio
G0	None	17.4 (15.5–19.3)	32.1 (27.4–41.2)	1.0
G3	Control	19.7 (17.9–21.7)	32.2 (28.1–40.1)	1.1
	0.1 g/l MPs	22.6 (20.3–25.0)	40.6 (35.3–50.1)	1.3
	1.0 g/l MPs	25.1 (22.4–27.9)	49.3 (42.4–61.4)	1.4
	10 g/l MPs	31.7 (28.5–35.2)	62.1 (53.0–79.1)	1.8
G6	Control	20.9 (19.2–22.9)	28.7 (25.9–33.7)	1.2
	0.1 g/l MPs	25.6 (22.8–28.6)	53.0 (45.2–67.0)	1.4
	1.0 g/l MPs	27.0 (23.8–30.4)	62.3 (51.9–82.4)	1.5
	10 g/l MPs	35.0 (31.5–38.7)	70.2 (58.2–93.9)	2.0
G0	None	17.4 (15.5–19.3)	32.1 (27.4–41.2)	1.0
G3	Control	19.7 (17.9–21.7)	32.2 (28.1–40.1)	1.1
	Insecticide only	27.7 (21.2–34.8)	44.2 (35.0–85.7)	1.6
	0.1 g/L Mixture	23.7 (21.6–26.0)	37.4 (33.0–45.4)	1.4
	1 g/L Mixture	28.6 (25.8–31.6)	52.7 (45.8–64.7)	1.6
	10 g/L Mixture	33.4 (30.4–36.4)	54.4 (48.4–65.5)	1.9
G6	Control	20.9 (19.2–22.9)	28.7 (25.9–33.7)	1.2
	Insecticide only	28.7 (25.9–31.4)	47.6 (42.1–57.0)	1.6
	0.1 g/l Mixture	27.5 (24.8–30.3)	48.2 (42.3–58.3)	1.5
	1 g/l Mixture	31.5 (28.4–34.6)	55.8 (48.8–68.3)	1.8
	10 g/l Mixture	37.5 (34.3–40.7)	59.6 (53.0–72.0)	2.1

In the treatments containing mixtures of MPs and insecticide, the KDT_50_ and KDT_95_ varied from 19.7 to 33.4 and 32.2 to 54.4 minutes, respectively. Similar trends were observed in G6, with KDT_50_ and KDT_95_ ranging from 21 to 38 and 29 to 60 minutes. The highest KDT values were noted in the 10 g/L mixture of G6, while the lowest was in the baseline (G0), indicating increasing mosquito tolerance with higher mixture concentrations. However, in mosquitoes from G6, the resistance ratios ranged from 1 to 1.9 in G3 and 1 to 2.1, suggesting a potential rise in mosquito tolerance to insecticides over generations and treatments.

### Mortality rate of adult mosquiotes after 24 hours

**Fig 2:**
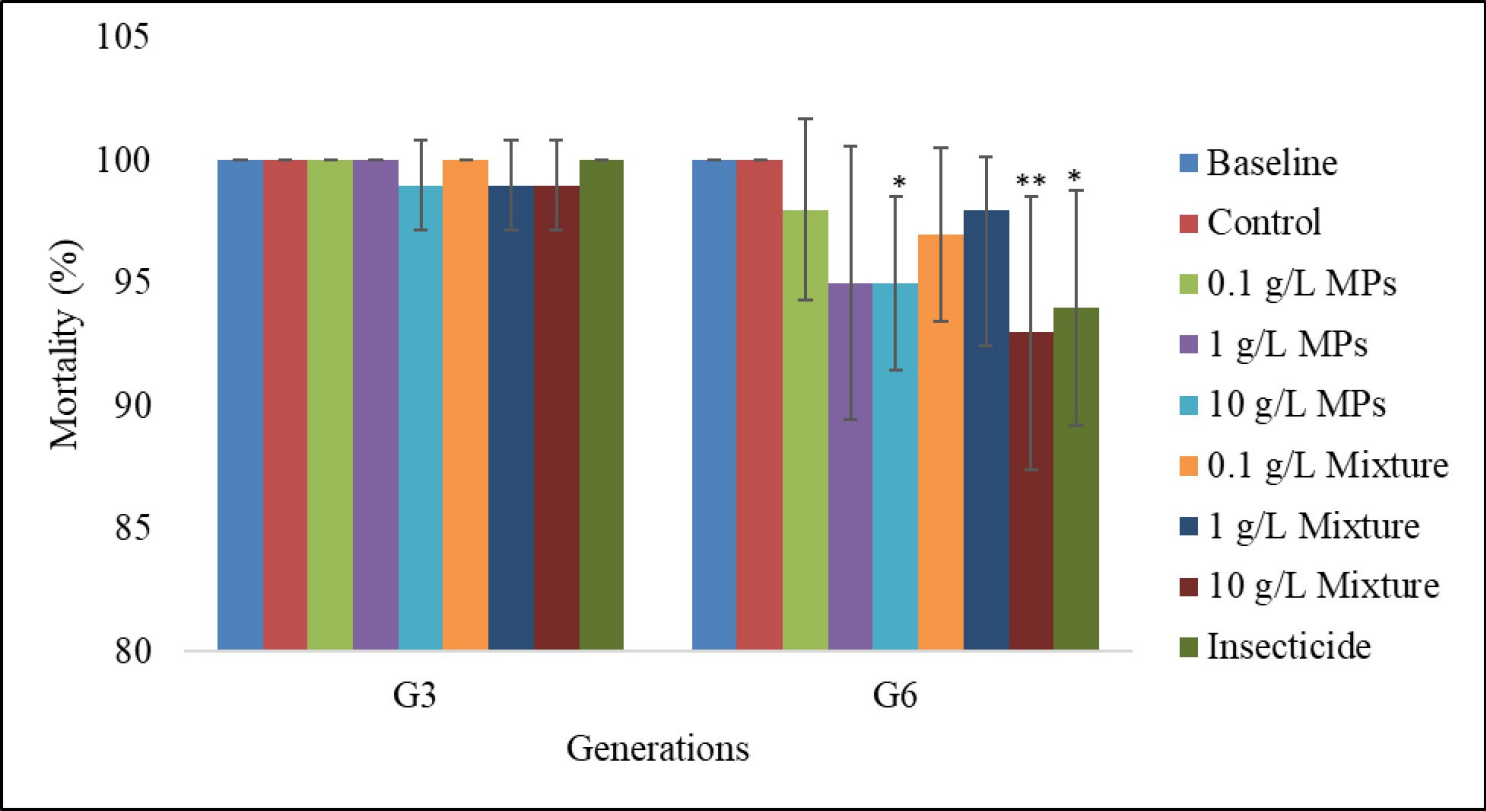
Mean mortality rates of female *An*. *gambiae* s.s. reared from larvae exposed to MPs and mixture. Error bars represent standard deviation. Asterisks denote significant differences between each treatment and the corresponding control (*P < 0.05, **P < 0.005, generalized linear model tests).

At 24 hours post-exposure to the insecticide, adult mosquitoes from the untreated baseline larvae (G0) exhibited a mortality rate of 100% (Fig 2). Up to generation 3 (G3), the mortality rates of adult mosquitoes from larvae reared in all treatments, including the control, remained consistently high (99–100%), with no significant differences observed between the MP treatments (F_(4,39)_ = 2.33, p = 0.075) or the MP-insecticide mixtures (F_(5,47)_ = 1.87, p = 1.12) when compared to the control. By generation 6 (G6), significant differences in mortality rates emerged between treatments, with adult mosquitoes from larvae reared in either MP treatments (F_(4,39)_ = 4.4, p = 0.005) or mixtures (F_(5,47)_ = 5.96, p < 0.0001) exhibiting lower mortality rates. The control group (100% mortality) showed significantly higher mortality compared to those exposed to 10 g/L MPs (p = 0.041), 10 g/L Mixture (p < 0.0001), and the insecticide-only treatment (p = 0.001) (Fig 2).

## Discussion

This study has demonstrated the potential for microplastics to adversely affect *Anopheles gambiae s*.*s*. by increasing their resistance to insecticides. Therefore, MPs in larval breeding sites could be contributing to the development of insecticide resistance in these important malaria vectors, which is a significant concern for environmental control programmes.

### Effects of MPs on survival and development time of mosquito larvae

When the first generation of *An*. *gambiae* s.s larvae were exposed to PET-MPs, a significant reduction in their survival rate and an increase in their development time was observed, with a more noticeable effect at higher MP concentrations. Similar decreases in larval survival rate and development have been shown following exposure to microplastics of other species of mosquito (e.g., [12]), demonstrating that at environmentally relevant concentrations, MP polymers can have negative effects on insect life history traits. Such harmful effects of MPs may result from their ingestion, accumulation, and subsequent obstruction and irritation of the gut, potentially delaying the development of larvae [13, 34]. Obstruction blocks food flow, while irritation refers to physical or chemical damage to the gut lining that might be caused by the toxic or abrasive properties of MPs, leading to inflammation or discomfort. Gut obstruction (or satiation) can also lead to reduced feeding rates, while gut irritation is often associated with induced inflammation and cell death.

There are previous studies however, that report no significant effect of exposure to MPs (e.g., 1–5.8 μm polystyrene microbeads) on the survival and development time of the mosquitoes *Ae*. *albopictus*, *Ae*. *aegypti*, *Cx*. *pipiens* and *Cx*. *tarsalis* [9, 35–37]. These inconsistencies are likely due to study-specific experimental parameters, including the species of mosquito, the stage of larval exposure, and the rearing temperature, together with the size, shape, concentration, and polymer of the MP. For example, we used MP fragments rather than microbeads and exposed *An*. *gambiae* s.s to these MPs at earlier larval stages and used slightly higher experimental temperatures than previous studies [9, 35–37]. Importantly, all of the experimental parameters we have used in this study are field-relevant, and reflect conditions that mosquito larvae would commonly be exposed to in the environment.

In the multigenerational experiment spanning six generations, the impact of PET on subsequent generations of mosquito larvae increased survival rate and development time, which was consistently observed across all treatment groups. Importantly, persistent exposure over subsequent generations appeared to induce some level of larval adaptation towards MP exposure, indicating the development of some degree of tolerance over generations. Similar phenomena have been documented in *Ae*. *Aegypti* following multigenerational rearing on hydrocarbon pollutants [18]. Continual exposure to MPs may have selected for larvae with a larger gut, which may have allowed the absorption of nutrients despite obstruction by MPs; in addition, a larger gut may have facilitated the elimination of MPs through egestion [12]. Biochemical and genetic responses to multigenerational exposure to MPs have also been reported in other invertebrates, including induction of oxidative stress mediator enzymes (*Caenorhabditis elegans*; [38]), overexpression of stress-mediating genes such as heat shock proteins and superoxide dismutase (*C*. *riparius*; [39, 40]), and increased expression of genes involved with the oxidoreductase process (*Chironomus riparius*; [17]). Mechanisms of adaptation to MPs in mosquitoes may influence traits beyond survival and development time, and potentially impact vector competence [17].

### Effects of MPs with insecticide on survival and development time of mosquito larvae

Exposure of larvae to mixtures of MPs with a sub-lethal dose of the insecticide lambda-cyhalothrin, led to significantly higher larvae survival rates and shorter development times compared to exposure of larvae to the insecticide alone. This suggests that MPs are capable of reducing the efficacy of insecticides on mosquitoes [13]. Inherent characteristics of MPs, e.g., large surface area, microporous structure, intermolecular van der Waals and electrostatic forces, facilitate the sorbtion of both polar and non-polar pollutants such as insecticides dissolved in water [41, 42], which are commonly utilized for mosquito larval control. Multigenerational exposure of larvae to mixtures of MPs and insecticide increased survival rates and decreased development time (compared to exposure to insecticide alone), suggesting selective pressure on traits that confer adaptive advantages to insecticide exposure [43–45]. Therefore, field-scale studies are now urgently required to quantify whether larval insecticides are less effective at controlling mosquitoes in breeding sites commonly polluted with MPs.

### Susceptibility of adult *An*. *gambiae* s.s. to insecticide treatments

The susceptibility to lambda-cyhalothrin was assessed in female adult *An*. *gambiae s*.*s*. (following multigenerational exposure of the larvae to MPs and mixtures of MPs with insecticide) at baseline (G0), third-generation (G3), and sixth-generation (G6), with an increasing insecticide tolerance seen across generations in those exposed to either MPs alone or the mixture treatments. Sub-lethal doses of insecticides can play a significant role in promoting resistance in insect populations by allowing survive during the initial exposure, which creates a selective pressure that favours those individuals with resistance traits. These traits are passed down to future generations, diminishing the effectiveness of the insecticide. This is likely the reason for the significantly lower mortality rate observed at G6, especially in cases where the larvae had been exposed to the insecticide mixed with the two higher MP concentrations (where there was greater potential for the insecticide to bind to the surfaces of MP and therefore effect the concentration that the larvae were being exposure to), indicating that prolonged larval exposure to MPs alone or mixed with insecticide may accelerate the development of insecticide resistance in adult mosquitoes. This is the first study demonstrating the selection of insecticide tolerance in adult mosquitoes after consecutive generations of larval exposures to varying concentrations of MPs. A similar effect was observed in *Aedes aegypti*, with increased tolerance to *Bti* larvicide after larvae had been reared on micropollutants like ibuprofen or benzo[a]pyrene over six generations [18]. Additionally, the development of tolerance to insecticide was demonstared in adult *An*. *gambiae* s.s. and *An*. *arabiensis* after long-term exposure to domestic waste pollutants such as hydrogen peroxide and soaps [46].

Long-term exposure to MPs could induce stress mediators such as *HSP*, *SOD*, catalase (CAT), acetylcholinesterase and *GST* during larval stages [17]. If these traits persist into adult stages, they might also mediate the stress caused by insecticide exposure in adults, potentially leading to insecticide resistance, e.g., overexpression of genes such as SOD and GST and HSP have been linked to insecticide resistance in mosquitoes [47, 48]. Selection of resistance to various insecticides at sub-lethal doses has been widely reported [44, 45, 49]; however, in addition to potentially reducing the effectiveness of larvicidal insecticides within breeding sites, MPs may also be contributing to the development of insecticide tolerance in both mosquito larvae and adults.

## Conclusion

This study has demonstrated that exposing *An*. *gambiae* s.s. larvae to PET-MPs with and without the addition of insecticide over six generations can affect survival rates, larval development time, and adult mosquito insecticide tolerance. Initially, MP-exposed larvae showed reduced survival rates and longer development times, but later generations adapted to any negative effects of being exposed to MPs. Mixing MPs with insecticide reduced the efficacy of the insecticide, which became more apparent over subsequent generatons of larvae, and was translated into increased insecticide tolerance in adult mosquitoes. These findings suggest that prolonged exposure of *An*. *gambiae* s.s. larvae to MPs may enhance their tolerance to MP pollution in the environment, with the potential for the interaction of MPs with insecticide leading to resistance in both larvae and adults.
